# The process of self-care in patients with heart failure after nurse-assisted remote patient monitoring: A qualitative longitudinal approach

**DOI:** 10.1016/j.ijnsa.2025.100426

**Published:** 2025-09-19

**Authors:** Signe Østrem, Anna Strömberg, Kari Hanne Gjeilo, Marianne Storm, Ingvild M. Morken

**Affiliations:** a Department of Public Health, Faculty of Health Sciences, University of Stavanger, 4036 Stavanger, Norway; b Department of Intensive Care, Stavanger University Hospital, Stavanger, Norway; c Department of Health, Medicine and Caring sciences and Department of Cardiology, Linköping University, Sweden; d Department of Public Health and Nursing, Faculty of Medicine and Health Sciences, NTNU – Norwegian University of Science and Technology, Trondheim, Norway; e Clinic of Cardiology, St. Olavs Hospital, Trondheim University Hospital, 7006 Trondheim, Norway; f Research Group of Nursing and Health Sciences, Research Department, Stavanger University Hospital, Stavanger, Norway; g Faculty of Health Sciences and Social Care, Molde University College, Molde, Norway; h Department of Quality and Health Technologies, Faculty of Health Sciences, University of Stavanger, Stavanger, Norway

**Keywords:** Content analysis, Heart failure, Longitudinal qualitative research, Remote patient monitoring, Self-care

## Abstract

**Background:**

Effective self-care, including symptom monitoring and treatment adherence, is essential for individuals with heart failure. It can enhance quality of life, improve survival, and reduce hospital admissions. The home is the primary setting where individuals with heart failure perform self-care. However, many struggle to recognize symptoms that indicate worsening of their condition. Remote patient monitoring allows nurses to track symptoms, weight, and blood pressure while supporting self-care behaviours that help prevent exacerbations. A clearer understanding of these interventions is needed, particularly regarding how they influence patients’ views on self-care over time.

**Objective:**

This study explored short- and long-term experiences of self-care among individuals with heart failure after a six-week, nurse-assisted remote patient monitoring intervention.

**Setting:**

Twelve participants were recruited from the intervention group in a randomised controlled trial. Clinical trial ID 301472.

**Methods:**

This study employed a qualitative longitudinal approach. Eleven semi-structured interviews were conducted at two time points: at time one, immediately after participants had completed the nurse-assisted remote patient monitoring intervention; and at time two, six months post-discharge. One participant did not respond to the second interview, resulting in 23 interviews in total. A qualitative content analysis was undertaken to explore the evolving self-care process over time.

**Results:**

‘Transition from digital dependence to independent self-care management’ emerged as the overarching theme describing participants’ experiences at both time points. This theme was illustrated by four subthemes identified at time one: (1) guidance to interpret symptoms and bodily signs; (2) establishing a daily routine in monitoring vital signs; (3) support for changes in lifestyle and medication adherence; and (4) sense of security. At time two, three subthemes were identified: (1) increased confidence in bodily awareness and symptom monitoring; (2) recognition of self-care routines; and (3) feeling in control due to prior feedback from a previous nurse navigator.

**Conclusions:**

Individuals with heart failure experienced self-care as an evolving process. This was demonstrated by their transition from reliance on digital support from nurses and the remote patient monitoring intervention to increased independence and proactivity in managing their self-care. There is an urgent need to respond to the pressures facing overburdened health-care systems. These findings highlight the importance of developing a digital environment that supports sustainable transitional care from hospital to home for individuals with heart failure.

**Registration:**

The main randomised controlled trial project, “eHealth@hospital-2-home," is registered under ClinicalTrials.gov ID: 301472. It was registered on 27.02.2023, with the first recruitment commencing on 03.05.2023.

**What is already known**

• Inadequate follow-up after hospital discharge can leave individuals with heart failure feeling unsafe and anxious, which may contribute to poor adherence to self-care and reduced quality of life.

• Early recognition of symptoms is essential in heart failure management, as it may help prevent condition progression.

• Remote patient monitoring interventions are a promising strategy for enhancing self-care management by providing individuals with heart failure access to real-time data and professional support.

Alt-text: Unlabelled box

**What this paper adds**

• This study demonstrates that a short-term, post-discharge remote patient monitoring intervention can support the development of sustainable self-care routines and enhance individuals’ sense of control in managing heart failure at home, with benefits that may be retained over time.

• The nurse-navigators in this study tailored their feedback and responses based on participants recorded symptoms and physiological measurements, illustrating how personalised remote care can reinforce effective self-care behaviours.

Alt-text: Unlabelled box

## 1 Introduction

Heart failure is a prevalent chronic condition, with an estimated 64.3 million individuals living with heart failure globally (Groenewegen et al., 2020). There has been a rise in the prevalence of heart failure over recent decades, driven by a combination of demographic shifts, and advancements in treatment. Key contributors to this increase include are an enhanced global life expectancy, which enables more individuals to reach an age where they are at risk of developing heart failure, better diagnostics, higher survival rate from conditions causing heart failure, such as myocardial infarction, hypertension and diabetes, and more advanced and effective heart failure treatment with medications and devices (Savarese et al., 2022).

Heart failure imposes a substantial symptom burden, necessitating comprehensive healthcare strategies. Effective management depends on recognising symptoms such as dyspnoea, fatigue, and fluid retention, as well as addressing coexisting comorbidities (Albert et al., 2015; Ambardekar and Thielen, 2023; McDonagh et al., 2023).

To alleviate this symptom burden, individuals must adhere to a demanding self-care regimen, which includes lifestyle modification and timely response to emerging symptoms (Malasinghe et al., 2019; Nordfonn et al., 2021). Approximately 25% of individuals with heart failure are readmitted shortly after discharge, with nearly 50% readmitted within six months leading to serious clinical, economic, and social consequences (Wideqvist et al., 2021). Adherence to self-care and medication regimens is essential to reducing hospital readmissions (Lee et al., 2018; Wu and Moser, 2018).

Self-care in chronic illness is defined as a process of maintaining health-promoting behaviours and managing illness (Riegel et al., 2012). This process comprises three distinct yet interconnected phases: maintenance, monitoring, and management (Riegel et al., 2012). Together, these phases represent a comprehensive approach involving ongoing health maintenance, symptom observation, and appropriate response when changes occur (Riegel et al., 2019).

The transition from hospital to home represents a critical and vulnerable period for individuals with heart failure, who often receive limited support from health services (Rosano et al., 2022; van Grootel et al., 2024). During this time, many experience difficulties with self-care and face challenges in adhering to treatment regimens, including medications and lifestyle modifications. These difficulties frequently contribute to increased morbidity and high readmission rates (Istianah et al., 2023; Patidar et al., 2021). Effective follow-up after hospital discharge can encourage early engagement in self-care and help slow disease progression (Longhini et al., 2025; Olofsson et al., 2025). Remote patient monitoring technologies offer a promising strategy for tracking symptoms and signs by providing real-time data and support to individuals with heart failure, potentially leading to improved health outcomes (Serrano et al., 2023).

### 1.1 Background

Managing heart failure at home requires a high degree of self-management, which includes the knowledge, skills, and competencies necessary for effective self-care (Riegel et al., 2016, 2012). This entails adopting health-promoting behaviours, such as following a specific diet, engaging in regular physical activity, and adhering to prescribed medication regimens. Monitoring often described as bodily awareness is also a key element of self-care, allowing individuals to track vital signs such as blood pressure and to recognise changes over time (Matarese et al., 2018). These practices are crucial for maintaining health and managing heart failure. Moreover, self-care demands the capacity and motivation to sustain these health-promoting behaviours consistently (Riegel et al., 2011).

Unfortunately, many individuals face challenges in distinguishing symptoms and signs that indicate worsening heart failure from those related to comorbidities or normal age-related changes (Aamodt et al., 2020; Okada et al., 2019). This difficulty can result in delays in seeking timely care (Okada et al., 2019). Additionally, individuals may adjust their lifestyle or daily routines in response to their perceived health status, which may impair their ability to recognise critical signs of decompensation (Jaarsma et al., 2021). Early recognition of symptoms in individuals with heart failure living at home is vital, as it may prevent the condition from progressing to a level that requires hospitalisation (Bui and Fonarow, 2012). The European Society of Cardiology recommends regular follow- by healthcare professionals to support optimal management (McDonagh et al., 2023). However, workforce shortages, an ageing population with complex comorbidities, and policies promoting shorter hospital stays have made traditional in-person follow-up increasingly difficult to sustain (World Health Organization, 2016). One potential solution to enhance follow-up and address these systemic challenges is the implementation of remote patient monitoring interventions.

Remote patient monitoring interventions may offer a valuable opportunity to actively engage individuals with heart failure in self-care management (Deckwart et al., 2023). These interventions facilitate communication between healthcare providers and individuals through various modalities, including audio, video, and text-based interactions (Malasinghe et al., 2019). Remote patient monitoring can track vital signs such as temperature, pulse rate, blood pressure, weight, and oxygen saturation directly from the user’s home over extended periods (Ko et al., 2023). This capability not only allows healthcare providers to assess health status remotely, but also enhances communication with users, thereby improving the overall management of self-care (Edmunds, 2019; Wartenberg et al., 2025). As a result, users of remote patient monitoring become more engaged in their care, as they can access their health data and better understand the effects of their lifestyle choices (Tagne et al., 2025).

However, a more comprehensive understanding of remote patient monitoring applications is needed particularly concerning their effects on users and their perspectives on self-care maintenance over time. By emphasising the need for longitudinal qualitative insight into self-care following a remote patient monitoring intervention, this study may address a critical gap in underexplored research (Nick et al., 2021; Walker et al., 2019). Additionally, by exploring the trajectory of change over time, this study may offer insights into the long-term impact of the remote patient monitoring intervention. This includes understanding how long the benefits persist and how much time patients need to establish self-care routines and build confidence after being discharged from the hospital (Saldaña, 2002). Therefore, this study aimed to explore the evolving nature of self-care over time by examining both short- and long-term experiences among individuals with heart failure following a six-week nurse-assisted remote patient monitoring intervention.

## 2 Methods

### 2.1 Design

This qualitative longitudinal study was conducted alongside a randomised controlled trial that evaluated the effect of a nurse-assisted remote patient monitoring intervention on self-care in individuals with heart failure and colorectal cancer, referred to as “eHealth@hospital-2-home” (Storm et al., 2024). A qualitative longitudinal approach offers a unique opportunity to gain nuanced insights into changes in participant perspectives over time, thereby facilitating a deeper understanding of the intervention's impact and sustainability (Audulv et al., 2023).

Philosophically, this study was grounded in a critical realist approach (Danermark et al., 2019; Sabau, 2024). Critical realism posits that reality is stratified, comprising both observable phenomena and underlying generative mechanisms that influence behaviour and experience, thus allowing processes to evolve (Danermark et al., 2019).

#### 2.1.1 Participants

The study population comprised 12 individuals with heart failure, nine men and three women. They were recruited from the intervention group of the “eHealth@Hospital-2-Home” trial, which included participants who received a nurse-assisted remote patient monitoring intervention as part of a sub-study focusing solely on individuals with heart failure. Participants in the sub-study were recruited prior to discharge from hospital wards at two university hospitals in Norway. Prior to the study, there was no established relationship or knowledge between the researchers and the participants.

Eligibility criteria in the “eHealth@Hospital-2-Home" trial included hospitalised individuals with symptomatic heart failure, such as dyspnoea at rest, pulmonary congestion, and elevated NT-proBNP levels >1000 pg/mL. Additional inclusion criteria were the ability to speak and write Norwegian and being over 18 years of age.

Exclusion criteria were the requirement for Left Ventricular Assist Device, being on a waiting list for heart transplantation, or having a life expectancy of less than six months. Individuals were also excluded if they had a severe mental illness or cognitive impairment, were planned for discharge to a nursing home, were participating in another intervention study, or were unable to stand independently on a weight scale.

**Fig. 1:**
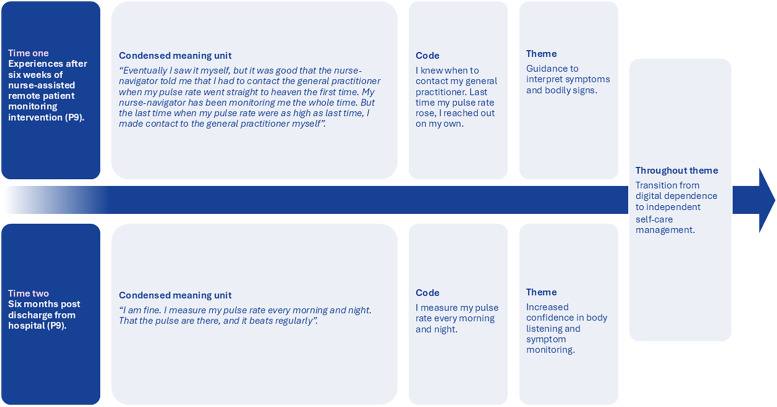
Flowchart for heart failure patients with a longitudinal qualitative study alongside the randomised controlled trial sub-study of “eHealth@Hospital-2-Home.

After consenting to participate in the trial and being randomised to the intervention group, participants were purposively selected using maximum variation sampling to ensure a broad representation across age and sex. Around week five of the intervention, the nurse-navigators contacted eligible participants by telephone to invite them to the qualitative study. The sample size was intentionally limited to 12 participants, which is considered adequate across multiple qualitative methodologies to obtain rich, in-depth data without redundancy (Malterud et al., 2016; Young and Casey, 2018). A flowchart detailing the longitudinal qualitative study alongside the heart failure sub-study within the “eHealth@Hospital-2-Home" trial is provided, see Fig. 1.

#### 2.1.2 The nurse navigators

Nurse navigators in a remote patient monitoring context, serve as the primary point of contact, interpreting data, responding to alerts, and supporting individuals in self-care. In this study, five registered nurses from two university hospitals in Norway functioned as nurse navigators. These nurses were experienced hospital professionals with the clinical skills required to guide individuals through complex care pathways, coordinate care across various health services, and provide education, emotional support, and follow-up care after hospital discharge via our remote patient monitoring intervention.

#### 2.1.3 The nurse-assisted remote patient monitoring intervention

This intervention is a non-acute, alert- and communication-based service operated by nurse navigators. It is a digital health service that monitors patient data and generates alerts when readings fall outside predefined thresholds. Additionally, it enables two-way communication between individuals and healthcare professionals via messaging or video calls (Storm et al., 2024). This nurse-assisted remote patient monitoring intervention used an existing software platform, Dignio Connected Care, which was accessible to patients via the MyDignio application and to nurse-navigators via the DignioPrevent application (Dignio, 2022). Together, MyDignio and DignioPrevent form a comprehensive remote care ecosystem that empowers patients to manage their own health under virtual supervision by the nurse navigators.

The intervention lasted six weeks after hospital discharge and aimed to monitor and support participants. During this period, individuals performed daily measurements, such as blood pressure, heart rate, and body weight, which were automatically transmitted to the Dignio Connected Care application via Bluetooth. In addition, participants completed a daily symptom checklist and a weekly well-being checklist. The daily measurements were visualised in the Dignio Connected Care application as a remote patient monitoring graph.

### 2.2 Data collection

Data were generated from two interviews conducted with each included participants, starting immediately after completing the six-week post-hospital discharge remote patient monitoring intervention (time one), and then at six months post-discharge (time two). Both interviews were collected by first author. One participant did not respond to the invitation for the second interview, resulting in a total of 23 interviews, 12 at time one and 11 at time two. Seventeen interviews were conducted in participants’ homes, while six took place in a location they selected. Semi-structured face-to-face interviews were conducted to address the study objectives and collect data (Brinkmann and Kvale, 2015).

Each interview was carried out by the first author, using a general interview guide at time one and a personalised interview guide at time two (Audulv et al., 2022; Hermanowicz, 2013), employing an audio recording device. The recordings were then transcribed verbatim.

The primary questions in both interviews at time one and time two are outlined in Table 1. In addition to these primary questions, several open-ended and follow-up questions were posed in both interviews, such as: "How did you experience that? ", "Have you had any similar experiences since the last interview?", and "Can you explain this further?”

**Table 1:** Illustration of the questions at time one and time two.

Interview guide illustration	
Time one	**Time two**
1. Could you describe your experience using the nurse-assisted remote patient monitoring intervention?	1. How is your life at present?
2. How do you describe self-care in your own words?	2. How is your life without the nurse-assisted RPM intervention?
3. How, if at all, has the remote patient monitoring intervention influenced self-care practices?	3. Reflecting on your time using the nurse-assisted RPM tool have you noticed any changes in your thoughts or experiences? For example, do you have new insights or experiences since you last used it?
4. How did you experience being followed up by a nurse-navigator?	4. How do you describe self-care today? In our previous interview you described your approach to self-care. Could you explain how you manage it now? Have you encountered any new experiences or adopted new strategies?
5. How did you experience using the remote patient monitoring tool during your transition from hospital to home?	5. Have you developed any new thoughts regarding being followed up by a nurse navigator?

The second interview guide was developed by reviewing the audio recordings and reading the transcript from the first interview. The questions for the six-month interview were based on Saldaña’s (2003) seven descriptive questions, with a focus on the participants’ responses from the initial interview and their experiences regarding self-care. This approach allowed for individual, in-depth interviews that explored the participants’ previous statements and examined any changes since the first interview. This helped us to explore changes in self-care experiences, influenced by the nurse-assisted remote patient monitoring intervention, over time. Data collection occurred between October 2023 and August 2024.

### 2.3 Data analysis

The empirical data from time one and time two were analysed separately, resulting in two distinct themes, consistent with the longitudinal themes approach outlined by Audulv et sl., (2023). Additionally, content analysis as described by Elo and Kyngäs (2008), was used to identify and categorise subthemes from both time one and time two. Furthermore, the longitudinal themes approach facilitated the abstraction and synthesis of our findings at both time points, culminating in an overarching theme that integrated the findings. By combining these two approaches (Audulv et al., 2022; Elo and Kyngäs, 2008), our data analysis supported us to describe the participants personal experiences and provide narrative insights into the factors that may affect the participants evolving self-care over time.

The first step in our data analysis involved organising the qualitative data, which included open coding, categorisation, and abstraction (Elo and Kyngäs, 2008). This data organisation was carried out twice, once for time one and once for time two. The purpose of this process was to describe the evolving phenomena of self-care among individuals with heart failure between time one and time two (Audulv et al., 2023). Additionally, we aimed to gain knowledge about the process of self-care for individuals with heart failure within a longitudinal thematic approach (Audulv et al., 2022). For an overview of the analysis process, please refer to Appendix 1.

All transcriptions were managed using NVivo 15 software (Lumivero, 2025). The first author conducted the coding in NVivo, while the other authors had access to the NVivo file and regularly attended analysis meetings. This collaborative effort was designed to ensure credibility and dependability throughout the analysis process.

### 2.5 Rigour

To ensure trustworthiness in our study, measures were taken to verify credibility, confirmability, dependability, and transferability (Lincoln and Guba, 1985). Credibility was established through verbatim transcription of the interviews with participants. Verbatim transcription is essential in enhancing the credibility of qualitative research by maintaining fidelity to the original dialogue and enabling thorough analysis. This process captures the nuances of language, context, and emotion that may influence the interpretation of a participant's responses (Korstjens and Moser, 2018).

To increase confirmability, the first author (SØ) and the last author (IMM) independently analysed the data (triangulation), and all authors discussed the coding, categorisation, and abstraction processes until consensus was reached. Rival explanations as well as researcher preunderstanding, beliefs and assumptions were discussed throughout the analysis.

Dependability was ensured by using the same standardised interview guide and posing identical questions at the first time point. Additionally, to address our complex research questions regarding self-care experiences, we developed a personalised interview guide for the second time point to gain a deeper understanding of self-care for patients with heart failure. This development also contributed to the credibility of the study. A detailed description of the analysis process at all stages is provided in Appendix 1 to support transferability. Furthermore, we reported important aspects of the study (research team, methods, context, findings, analysis, and interpretation) and included an audit trail to facilitate the judgment of trustworthiness.

### 2.6 Ethical considerations

Ethical approval was granted by the Regional Ethics Committee for Medical and Health Research Ethics (REC ID: 556114), and we adhered to the World Medical Association Declaration of Helsinki for ethical research involving human participants (World Medical Association, 2024), as well as the principles of the General Data Protection Regulation. Each participant was required to sign an informed consent form and had the right to withdraw from the study at any time.

Throughout the study, the authors consistently reflected on and acknowledged that their pre-understanding and professional experience might have influenced the data collection, analysis, and interpretation processes. Their established expertise in heart failure research, along with their awareness of the challenges faced by heart failure patients in managing self-care in daily life, was carefully considered.

## 3 Results

### 3.1 Patient characteristics

The data from 12 participants resulted in a median interview length of 36 minutes (range: 14–150 minutes). The mean age of the participants was 68 years (range: 49–82 years). Three participants were women, and eight were married or had a partner. Ten participants were newly diagnosed with heart failure, and the average ejection fraction was 28%, ranging from 15% to 50%. Regarding comorbidities, eight participants had more than three comorbidities. The most common comorbidities were hypertension and diabetes, with six participants in both groups.

#### 3.1.1 Overarching theme and related themes

The data analysis revealed that the participants underwent a social process, beginning with their reliance on monitoring their vital signs and symptoms through remote patient monitoring and receiving feedback from nurses to manage self-care effectively and independently. "*Transition from digital dependence to independent self-care management*" emerged as the overarching theme and comprised the following four sub-themes identified at time one: "Guidance to interpret symptoms and bodily signs," "Establishing a daily routine in monitoring vital signs", "Support for changes in lifestyle and medication adherence," and "Sense of security." Additionally, the following three sub-themes emerged at time two: "Increased confidence in bodily awareness and symptom monitoring," "Recognition of self-care routines," and "Feeling in control due to prior feedback from the nurse-navigator".

**Fig. 2:**
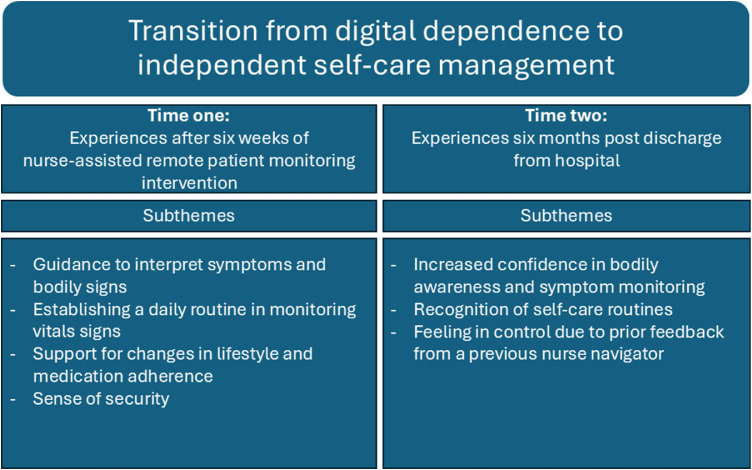
Illustration of the longitudinal theme and sub-themes at time one and time two.

Furthermore, our sub-themes are illustrated in Fig. 2. The four sub-themes identified at time one and the three sub-themes identified at time two are presented first, followed by the longitudinal overarching theme.

### 3.2 Experiences at time one, immediately following six weeks of nurse-assisted remote patient monitoring

#### 3.2.1 Guidance to interpret symptoms and bodily signs

During the remote patient monitoring intervention, participants received personalised guidance through the monitoring system and from the nurse navigator. Many participants reported that remote patient monitoring, in collaboration with the nurse navigator, helped identify bodily signs and symptoms that they had previously been unable to detect independently. Throughout the intervention, participants struggled to identify symptoms such as a high pulse rate and weight gain. However, as the intervention progressed, many participants began actively monitoring their vitals and were able to independently interpret their remote monitoring graphs. One participant stated:

“*Eventually, I saw it and managed it myself, but it was very helpful that the nurse navigator told me to contact the general practitioner the first time my pulse skyrocketed*”(P9).

The use of a symptom checklist within the remote patient monitoring intervention may have further helped participants become more aware of symptom registration, as highlighted by a participant:

“*Completely unconsciously, you think about it (symptoms). You are repeatedly told what to watch for when answering the questions (daily symptom list). Whether it’s swollen legs, chest pain, things like that*” (P3).

#### 3.2.2 Establishing a daily routine in monitoring vital signs

Immediately after completing the intervention, most participants stated that they would continue to monitor their vital signs independently. One participant remarked:

“*I have lived my whole life totally uninterested in my own body weight. But now I must buy a weighing scale so I can keep my weight under control*" (P4). Many participants reported establishing a new routine by performing daily measurements, and they indicated that there was no difficulty in conducting these measurements in practice. However, one participant noted, "*Blood pressure every day would be spasmodic*” (P10)..

#### 3.2.3 Support for changes in lifestyle and medication adherence

Several participants became more conscious of their medication adherence, made healthier dietary choices, or focused more on physical activity. While they may have had a healthy lifestyle prior to the remote patient monitoring intervention, they made minor yet beneficial individual changes to their lifestyles and daily routines as a result of the intervention and feedback from the nurse navigator. One participant shared:

"*I lost 10 kg when I was hospitalised, and when I got home, I decided I would not* let *myself get that heavy again. So, I have changed my diet to smaller portions*" (P4). The same participant also said, "*Remote patient monitoring gave me a new routine that I never had before*: a *routine about taking my medication* at *the same time, every day*" (P4).

Additionally, participants with comorbidities mentioned that this remote patient monitoring intervention also helped them manage their other chronic conditions, such as attention deficit hyperactivity disorder and diabetes.

#### 3.2.4 Sense of security

Participants described feeling safe and supported at home due to the intervention, largely because of the support from the nurse navigator at time one. The nurse navigator reached out when necessary, provided thoughtful responses to inquiries, and took participants' concerns seriously. One participant remarked: "*Without the comments from my nurse navigator and the close monitoring, the remote patient monitoring would not have provided the same sense of security*" (P9). Moreover, many participants described the nurse-assisted remote patient monitoring intervention as a form of supervision conducted within the comfort of their own homes, which contributed to their sense of security. One participant explained:

"*I would say that it was a tremendously good and safe way to be followed up on. You* could *say that you felt taken care of, in that you didn’t just go home to be on your own. You knew you were sent home without* leaving *all the people at the hospital, because you knew there was someone watching you every day through the remote patient monitoring. It felt like a small doctor’s visit e*ach *morning when you hit send on your daily measurements. You knew someone was watching what you had typed in” (P3).*

Furthermore, participants described monitoring their vitals via graphs on a tablet device and registering changes in collaboration with the nurse navigator. This process increased their sense of security. One participant shared:

"*I experienced the remote patient monitoring intervention like a lifeline. I could see that my vitals were where they should be, and this increased my sense of security at home*" (P5)..

### 3.3 Time two, six months post-discharge

#### 3.3.1 Increased confidence in bodily awareness and symptom monitoring

When participants reflected on the first 6 weeks of the nurse-assisted remote patient monitoring intervention at time two, some reported that they no longer had the same tools or overview to monitor and manage their symptoms as they did during the intervention period. Others expressed that they had learned to monitor their bodily signs and symptoms independently without intervention. By monitoring their symptoms, participants highlighted the importance of tracking weight gain or loss, controlling their blood pressure when symptoms appeared, or manually checking their heart rate by palpating the carotid artery. Many participants mentioned that they continued to monitor their symptoms and bodily signs, making it an almost daily habit, either consciously or unconsciously, at time two. One participant at time two described their independent monitoring as follows:

"*Yes, it will probably always be in the back of your mind. When you get any of the symptoms, you probably recognise* them *more than you did before. You must, that’s for sure. So, you are a little aware of it all the way. Without it doing anything to everyday life*" (P3)..

#### 3.3.2 Recognition of self-care routines

Due to the nurse-assisted remote patient monitoring intervention, participants’ monitoring behaviours and awareness varied at time two. What they specifically monitored or became more aware of was influenced by the nurse navigator during the intervention, and they continued to recognise the importance of careful monitoring and awareness at time two. The individual feedback provided by the nurse navigator varied, and one participant shared:

"*I am glad that the nurse navigator gave me feedback, straight away. It’s that feedback I got back then that helps me today*" (P5).

The individual feedback provided by the nurse navigator included minor advice regarding lifestyle changes and self-care management. This covered daily activity, maintaining a good diet, and the importance of taking medication as directed. One participant already had an active lifestyle but needed to make dietary changes. The remote patient monitoring, together with the nurse navigator, provided specific, individual advice to each participant. This participant said:

"*A consequence of the remote patient monitoring is that I’m probably more concerned with what I eat and what I do to keep my body in shape, including fluids, food, and exercise. I kind of have to take care of myself, and it makes me more aware of what I do. Eat fewer snacks, increase my exercise and activity in* some *way. Because I’ve been extremely focused on being a coach for my kids. But now it’s that I’m squeezing in a walk, before or after training, right? And especially what I said a little earlier, that you become aware* that *what you do at home matters*" (P5).

Moreover, participants experienced the need to adapt and accept a new lifestyle due to their heart failure. This involved slowing down their daily activities at work and in their personal lives to reduce their workload and make life more manageable.

#### 3.3.3 Feeling in control due to prior feedback from a previous nurse navigator

The ongoing contact and regular feedback from the nurse navigator during the remote patient monitoring intervention contributed to a sustained sense of safety at time two.

"*During that period after hospitalisation, that’s probably when you are most vulnerable. It’s right after you are discharged that you feel most uncertain and such. And after some time, at least for me, I got the answers I wanted. And then the sense of security comes. So, when I finished the remote patient monitoring intervention, I felt that, yes, I have received the follow-up I needed, and I felt secure*" (P4).

One participant stated that he was less worried as a result of the remote patient monitoring intervention and the feedback he received from the nurse navigator at time two:

"*The nurse navigator told me that I should not worry about my Implantable Cardioverter Defibrillator, even if I think it’s uncomfortable. She said that it was normal and that I really didn’t have to worry about my Implantable Cardioverter Defibrillator. That feedback helps me today, and I remember that I really don’t need to worry and there’s nothing wrong* with *experiencing this sense of an uncomfortable feeling that I have*" (P5).

### 3.4 Overarching theme: Transition from digital dependence to independent self-care management

**Fig. 3:**
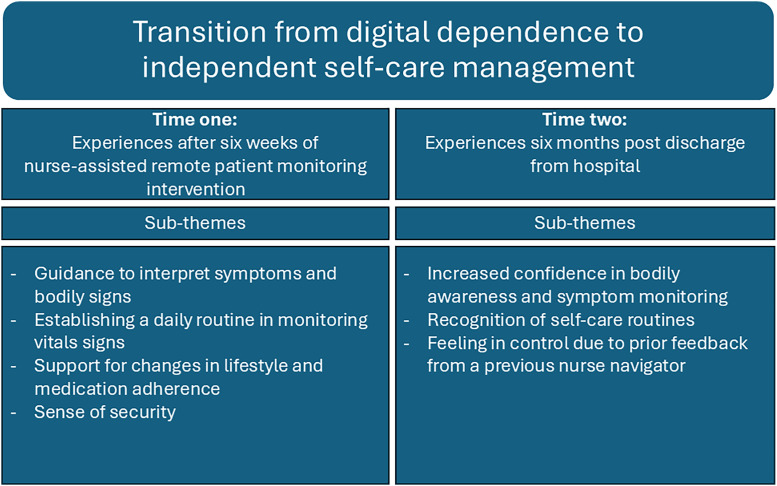
The evolving process of self-care management between time one and time two for one of the participants in this study.

By combining and analysing the two data sets from time one and time two, our overarching theme, "*Transition from digital dependence to independent self-care management,*” emerged. This theme describes how, at time one, participants initially relied on the nurse-assisted remote patient monitoring intervention and the nurse navigator’s feedback, but by time two, they had transitioned to managing their self-care independently. See the example in Fig. 3, which illustrates the self-care process that occurred between time one and time two.

At time one, the participant described in Fig. 2 experienced guidance to interpret bodily signs by learning to measure her heart rate through the nurse-assisted remote patient monitoring intervention. She also learned how to monitor her own measurements by using the remote patient monitoring graph to register a high heart rate independently. Additionally, she contacted the general practitioner herself before the nurse navigator reached out.

By time two, the same participant expressed increased confidence in monitoring her heart rate by palpating the carotid artery independently, without the support of the nurse-assisted remote patient monitoring intervention. Moreover, this participant described an increased awareness of self-care practices, measuring her heart rate every morning and night. She also reported feeling fine doing so, and by doing this, she felt in control of her own health at time two.

This participant’s experience of self-care had a specific focus on monitoring her heart rate. Throughout the nurse-assisted remote patient monitoring intervention, many participants described similar experiences, with a specified focus on self-care and what they needed to do to manage it. This focus on self-care varied among participants and was often a minor practice. These self-care practices were guided and supported by the nurse navigators during the intervention. The type of guidance and support participants received from the nurse navigator varied based on the background of the registered health data and deviations in the daily registered measurements.

## 4 Discussion

This study aimed to explore the experiences of patients with heart failure regarding self-care at two time points. Our findings indicated that participants engaged in a dynamic self-care process between these two time points. The cooperation and assistance provided by the nurse navigator during the nurse-assisted remote patient monitoring intervention were crucial for participants to engage in their self-care both during and after the intervention. The remote patient monitoring intervention enabled the nurse navigator to tailor support to participants' needs by registering and responding to deviations in their measurements. Our findings are consistent with previous studies that demonstrate the benefits of nurse-assisted remote patient monitoring in helping patients adhere to treatment and manage their self-care (Huang et al., 2024, 2022).

Moreover, our findings showed that the nurse-assisted remote patient monitoring intervention initially guided participants in assessing their symptoms and bodily signs. Over time, they became more confident in monitoring their health data, supported by the nurse navigator and the remote patient monitoring graphs. Regular feedback and open, two-way communication with the nurse navigator were key to this process. These experiences align with the findings of other studies that highlight how remote patient monitoring interventions can create opportunities for patient education, enhance symptom awareness, and facilitate the early detection of deterioration (Aamodt et al., 2020; Ekstedt et al., 2023; Michard and Saugel, 2024; Serrano et al., 2023; Wahyuni et al., 2023).

A new daily routine for monitoring vitals was established for participants during the nurse-assisted remote patient monitoring intervention. Many participants reported gaining increased knowledge and a better understanding of their chronic condition by monitoring their measurements. At the same time, they also received regular feedback from the nurse navigators on their measurements, even if the measurements were stable. This feedback, despite stable measurements, may have influenced the established routine of monitoring vitals due to the expectation set by the nurse navigator. Moreover, performing daily measurements may have been beneficial as it potentially increased participants' health literacy and their understanding of how to monitor their vitals, which in turn could enhance their self-care management (Dinh and Bonner, 2023). The significance of daily monitoring of vitals is also emphasised by the guidelines for heart failure management (McDonagh et al., 2023).

Some participants experienced those minor changes in lifestyle and medication adherence positively impacted their health. Due to the nurse-assisted remote patient monitoring intervention, participants were able to identify changes early in the remote patient monitoring graphs and actively adjust their medication or lifestyle to prevent worsening symptoms. Other studies emphasise the impact that nurse-assisted remote patient monitoring interventions can have on preventing deterioration through minor, but impactful adjustments in lifestyle and medications (Tan et al., 2024), such as adjustments to diuretics (Teleanu et al., 2025).

Furthermore, the nurse-assisted remote patient monitoring intervention provided participants with a sense of security at time one. Initially, they managed their heart failure at home with supervision and support from the nurse-assisted remote patient monitoring intervention. The shared health data between the participants and nurse navigators enhanced this sense of security. Other studies support our findings, which highlight the central role that nurse navigators play in nurse-assisted remote patient monitoring interventions (Ekstedt et al., 2023; Wathne et al., 2024).

At time two, our findings were similar to those at time one. The main difference at time two is that the findings reflect how participants maintained their self-care without the nurse-assisted remote patient monitoring intervention. At time two, participants had increased confidence in body listening and symptom monitoring. Some participants continued to monitor their vitals and bodily signs or symptoms independently. Moreover, they were aware of which self-care routines were relevant for them, thanks to the feedback they had received from the nurse navigators during the nurse-assisted remote patient monitoring intervention. Finally, many participants reported feeling in control of their chronic illness at time two. This sense of control is unfortunately not often experienced by patients with heart failure, who often report feelings of being overwhelmed and a lack of support in managing their chronic illness (Koontalay et al., 2024).

Our longitudinal findings align with previous studies that highlight the importance of symptom monitoring (Jaarsma et al., 2021), access to health data (Serrano et al., 2023) and receiving individualised counselling from a nurse navigator to foster an active self-care process (Serrano et al., 2023). Improving patients’ understanding and perception of their illness is crucial for promoting effective self-management. Furthermore, access to a nurse-assisted remote patient monitoring intervention may support patients by providing relevant knowledge of self-care behaviours and increasing their ability to manage their treatment and symptoms (Huang et al., 2022; Serrano et al., 2023). This transition from dependency to independence in self-care illustrates a self-care process that developed over time and may be similar to how Riegel et al. (2016) describe the self-care process.

### 4.1 Strengths and limitations

To our knowledge, this is the first study to provide qualitative longitudinal insights into the experiences of patients with heart failure regarding self-care after participating in a nurse-assisted remote patient monitoring intervention. The combination of a longitudinal approach with qualitative methods, using the content analysis approach of Elo and Kyngäs (2008), along with standard interviews at time one and a personalised interview at time two, to capture participants’ experiences and development over time, constitutes the most significant strength of this study. These elements facilitate a comprehensive understanding of the nuanced self-care processes that patients engage in over time.

However, this study has some limitations. Notably, the exclusion of patients who did not speak Norwegian may have limited our recruitment numbers. Additionally, the recruitment process did not yield so many female participants, and no individuals from ethnic minority backgrounds, which may have further restricted the diversity of perspectives represented in the study. Moreover, the main study, ehealth@hospital-2-home (Storm et al., 2024), had strict inclusion criteria, which may have introduced a selection bias in our study. The majority of our participants were newly diagnosed with heart failure, and their mean age was lower than the overall heart failure population in Norway (Norwegian Institute of Public Health., 2021). Furthermore, we did not have information about self-care in the control group of the randomised controlled trial, namely, those who had received standard care during the transition from hospital to home in the same period. Additionally, the transcripts, originally recorded in Norwegian, were translated into English by the first author, who is a non-native English speaker. This translation process may have introduced potential biases or inaccuracies in representing participants’ original narratives. In summary, while this study provides valuable insights into the self-care experiences of patients with heart failure after a remote patient monitoring intervention, it is essential to consider these strengths and limitations when interpreting the findings and their implications for future research and practice.

### 4.2 Implications for nursing practice

This study has important implications for healthcare systems, particularly in developing environments that support a sustainable transition from hospital to home. It is necessary to understand the impact of remote patient monitoring interventions on nursing workflows and explore strategies for integrating these interventions into existing healthcare systems.

## 5 Conclusion

In this study, participants described an evolving self-care process. The nurse-assisted remote patient monitoring intervention facilitated collaboration between nurse navigators and patients, enabling individuals with heart failure to engage with and actively monitor their health data and self-care over time. As a result, many participants became more proactive and aware of managing their self-care. The findings underscore the crucial role of nurse navigators in supporting patient self-care management. Our longitudinal findings illustrate that participants were initially dependent on the nurse-assisted remote patient monitoring intervention; however, by time two, many described increased confidence in managing their self-care independently. This suggests that nurse-assisted remote patient monitoring interventions have the potential to enhance users’ self-care and their ability to manage heart failure.

## Funding

This work was conducted in parallel with a randomized controlled study involving a nurse-assisted RPM intervention (‘Nurse-assisted eHealth service from hospital to home: Ameliorating the burden of treatment among patients with noncommunicable diseases’ [Funding No. 301372]). Moreover, this sub-study was funded by the Dam Foundation (No. 2023/FO426613).

## CRediT authorship contribution statement

**Signe Østrem:** Writing – original draft, Visualization, Validation, Methodology, Formal analysis, Data curation, Conceptualization. **Anna Strömberg:** Writing – review & editing, Methodology, Formal analysis, Conceptualization. **Kari Hanne Gjeilo:** Writing – review & editing, Methodology, Formal analysis, Conceptualization. **Marianne Storm:** Writing – review & editing, Methodology, Formal analysis, Conceptualization. **Ingvild M. Morken:** Writing – review & editing, Writing – original draft, Visualization, Validation, Supervision, Methodology, Formal analysis.

## Declaration of competing interest

The authors declare the following financial interests/personal relationships which may be considered as potential competing interests: Signe Ostrem reports financial support was provided by Stiftelsen DAM. Marianne Storm reports financial support was provided by Norwegian Research Council. If there are other authors, they declare that they have no known competing financial interests or personal relationships that could have appeared to influence the work reported in this paper.

## Appendix Supplementary materials

Image, application 1

Image, application 2
