# Erratum to “The process of self-care in patients with heart failure after nurse-assisted remote patient monitoring: A qualitative longitudinal approach” [International Journal of Nursing Studies Advances 9 (2025) 100426]

**DOI:** 10.1016/j.ijnsa.2025.100453

**Published:** 2025-11-19

**Authors:** Signe Østrem, Anna Strömberg, Kari Hanne Gjeilo, Marianne Storm, Ingvild M. Morken

**Affiliations:** a Department of Public Health, Faculty of Health Sciences, University of Stavanger, 4036 Stavanger, Norway; b Department of Intensive Care, Stavanger University Hospital, Stavanger, Norway; c Department of Health, Medicine and Caring sciences and Department of Cardiology, Linköping University, Sweden; d Department of Public Health and Nursing, Faculty of Medicine and Health Sciences, NTNU – Norwegian University of Science and Technology, Trondheim, Norway; e Clinic of Cardiology, St. Olavs Hospital, Trondheim University Hospital, 7006 Trondheim, Norway; f Research Group of Nursing and Health Sciences, Research Department, Stavanger University Hospital, Stavanger, Norway; g Faculty of Health Sciences and Social Care, Molde University College, Molde, Norway; h Department of Quality and Health Technologies, Faculty of Health Sciences, University of Stavanger, Stavanger, Norway

DOI of original article: < 10.1016/j.ijnsa.2025.100426>

The publisher regrets that the Fig. 3 was mistakenly included in the place of Fig. 1 and that Fig. 2 was mistakenly included in the place of Fig. 3.

**Fig. 1:**
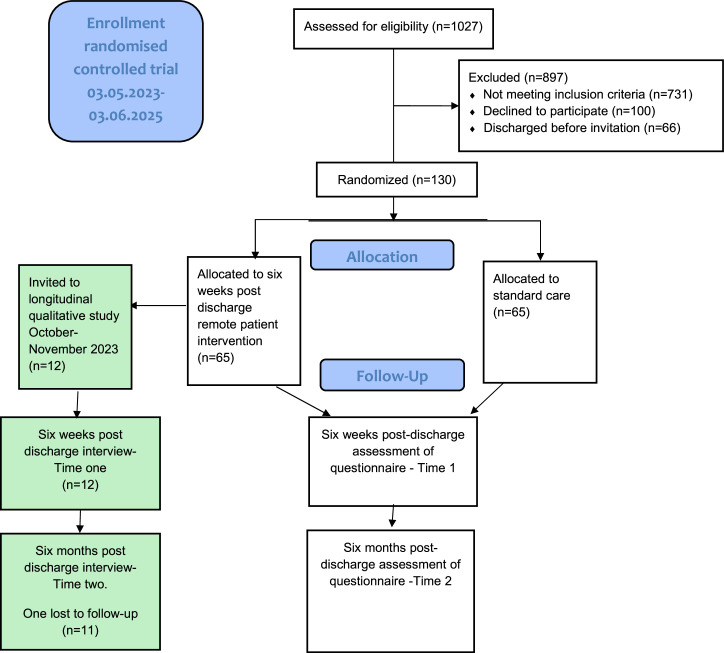
Flowchart for heart failure patients with a longitudinal qualitative study alongside the randomised controlled trial sub-study of “eHealth@Hospital-2-Home.

**Fig. 3:**
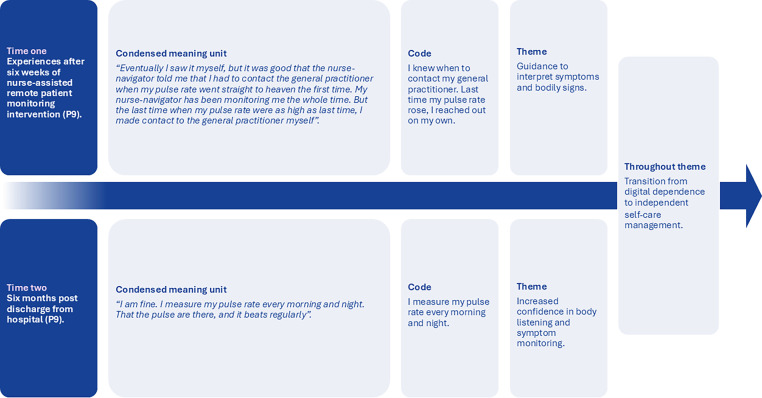
The evolving process of self-care management between time one and time two for one of the participants in this study.

The corrected Fig. 1, Fig. 3 are attached to this erratum.

The publisher would like to apologise for any inconvenience caused.

