# Supplementary material for: The process of self-care in patients with heart failure after nurse-assisted remote patient monitoring: A qualitative longitudinal approach
Source: Int J Nurs Stud Adv. 2025 Sep 19;9:100426. doi: 10.1016/j.ijnsa.2025.100426 (PMC12607116; doi:10.1016/j.ijnsa.2025.100426)
Supplement: Supplementary file 2 [file mmc2.docx]

| **Time one** | | | |
| --- | --- | --- | --- |
| Organising: | Code: | Sub-theme: | Overarching theme: |
| *“After about two weeks, I noticed that my weight had begun to increase- from 72 to 74 kg. Rather than immediately contacting my busy GP, I waited, thinking that the gain was minor. However, it was probably the rapidity of the increase that prompted the nurse navigator and doctors to intervene. I was prescribed an extra half-dose of diuretic for two days. I also received a message warning me that I had been drinking excessively, which surprised me because I had not expected to consume so much—even though I love to drink. In retrospect, had I waited another week, my weight might have increased even more” (P5).* | I noticed that my weight started to increase. I observed this change, and I suspect that the rate of this increase prompted the nurse-navigator to react. | Guidance in interpret symptoms and bodily signs. | Transition from digital dependence to independent self-care management. |
| *“Probably unconsciously, you consider your symptoms when you complete the daily checklist. This process informs you about what to watch for—for example, swollen legs, heart palpitations, or chest pain” (P3).* | Perhaps subconsciously, you begin to notice or reflect on your symptoms. | Establishment of a daily routine in monitoring vitals. |  |
| *“I stopped consuming the large volumes (of liquid) that I once did. I realized that I was probably drinking twice as much as I should have. When I observed the effects of this (behaviour), I began to feel better” (P5).* | Stopped consuming the large volumes of liquid. | Facilitating changes in lifestyle and medication adherence. |  |
| *“The greatest benefit I have experienced is the calm; I no longer have to wonder how it (my condition) is now” (P2).* | The greatest benefit is the calm. | Sense of security. |  |
| **Time two** | | |  |
| *“I still keep an eye on the weight and swelling in my body” (P8).* | Keep an eye on weight and swellings. | Increased confidence in body listening and symptom monitoring. |  |
| *“I've been so well informed, so I just stick to what I've done. There was a lot of routine, but I believe that to accomplish what I need to ,I must have some routines in my everyday life” (P2).* | I stick to what I’ve done, to my routines. | Awareness of self-care routines. |  |
| *"I pay attention to my pulse, and that is essentially it. Perhaps the most modest consequence of the intervention is that it increases awareness of proper self-care, which in turn motivates one to practice it" (P3).* | Awareness of proper self-care, which in turn motivates one to practice it. | Being in control due to feedback from a previous nurse navigator. |  |
